# COVID-19 Associated Coagulopathy Resulting in Cerebral Venous Thrombosis and Pulmonary Embolism

**DOI:** 10.7759/cureus.19602

**Published:** 2021-11-15

**Authors:** Zaira Abbas, Ali Chaudhary

**Affiliations:** 1 Stroke Medicine, Blackpool Teaching Hospitals, Blackpool, GBR

**Keywords:** d-dimer, coagulopathy, cerebral venous thrombosis cvt, pulmonary embolism (pe), covid 19

## Abstract

Venous stroke is an infrequent complication of severe acute respiratory syndrome coronavirus 2 (SARS-CoV-2) disease. Coronavirus disease 2019 (COVID-19) acts as a causative factor for thromboembolic events such as pulmonary embolism (PE), deep vein thrombosis (DVT), stroke (ischemic or hemorrhagic), and myocardial infarction. We report a case of cerebral venous thrombosis (CVT) following severe COVID-19 infection, with co-incidence of pulmonary thromboembolism. A 39-year-old English lady presented with fever and cough; subsequently, she was diagnosed with COVID-19 and was managed in the high dependency unit (HDU) due to the severity of symptoms; she received dexamethasone and tocilizumab. Her condition improved and she was discharged, but presented again after 15 days due to headache and left-sided weakness. Her neurological examination confirmed nystagmus, past pointing, and dysdiadochokinesia positive on the left side. Initial blood investigations showed D-dimer being raised at 1875 ng/ml. Head CT venogram reported evidence of thrombus in the superior sagittal sinus, left transverse sinus, and inferior sagittal sinus consistent with venous sinus thrombosis. She also underwent CT pulmonary angiogram (CTPA) which revealed lingular acute segmental PE and patchy ground-glass shadowing throughout both lung fields, confirming recent infective COVID-19 changes. She was started on a therapeutic dose of dalteparin (low-molecular-weight heparin). Luckily she made a good recovery from her neurological symptoms. Like this case and many other reported cases, COVID-19 acts as an independent risk factor for increased coagulopathy. Clinicians should maintain a high index of suspicion for CVT to aid in timely diagnosis and prompt treatment to save lives.

## Introduction

Coronavirus disease 2019 (COVID-19) is predominantly a respiratory disease caused by severe acute respiratory syndrome coronavirus 2 (SARS-CoV-2). Respiratory symptoms range from asymptomatic infection to severe pneumonia [1]. In fact, approximately 5% of COVID-19 patients suffer severe symptoms and often develop acute respiratory distress syndrome (ARDS) and systemic inflammatory response syndrome (SIRS). It can certainly affect other organs in addition to the original site of infection, causing multiple organ dysfunction/failure with high mortality (case fatality rate 49% in critical cases) [2]. Cardiovascular, renal, gastrointestinal, and neurological involvement are all examples of extra-pulmonary involvement [3]. It is noteworthy that many articles were found to have reported neurological manifestations of the COVID-19 infection, whereby 36.4% of patients had neurological symptoms and those with severe infection were also sometimes connected with strokes and coagulopathy [4]. Headache, myalgia, seizures, encephalopathy, encephalitis, encephalomyelitis, neuropathies (Guillain-Barré syndrome), dysosmia, dysgeusia, and stroke are some of the neurological abnormalities that have been found in various reports [5]. It is also established that COVID-19 acts as a causative factor for thromboembolic events such as pulmonary embolism (PE), deep vein thrombosis (DVT), stroke (ischemic or hemorrhagic), and myocardial infarction [6]. The increased levels of circulating pro-thrombotic micro particles such as antiphospholipid antibodies [7], fibrinogen, and D-dimer found in COVID-19 patients imply that this virus is playing a significant role in instigating a pro-thrombotic situation. The incidence of pulmonary thrombo-embolism based on CT pulmonary angiogram (CTPA) is found to be 30%, irrespective of the presence of clinical suspicion for PE in COVID patients [8].

Given the increased risk of thrombosis in SARS-CoV-2 patients, it is not surprising that the number of reports on cerebral venous thrombosis (CVT) and PE in the context of COVID-19 is increasing in the literature [9].

We report the case of a 39-year-old female who presented with CVT post severe COVID-19 Infection with co-incidence of PE. We describe details of clinical presentation, pro-thrombotic workup, neuroimaging findings, treatment, and outcome. This case is important because of the association between COVID-19 and thrombo-embolic phenomenon. It highlights the importance of considering CVT in the differential diagnosis in the context of previous history of COVID, especially in atypical presentations.

## Case presentation

**Figure 1:**
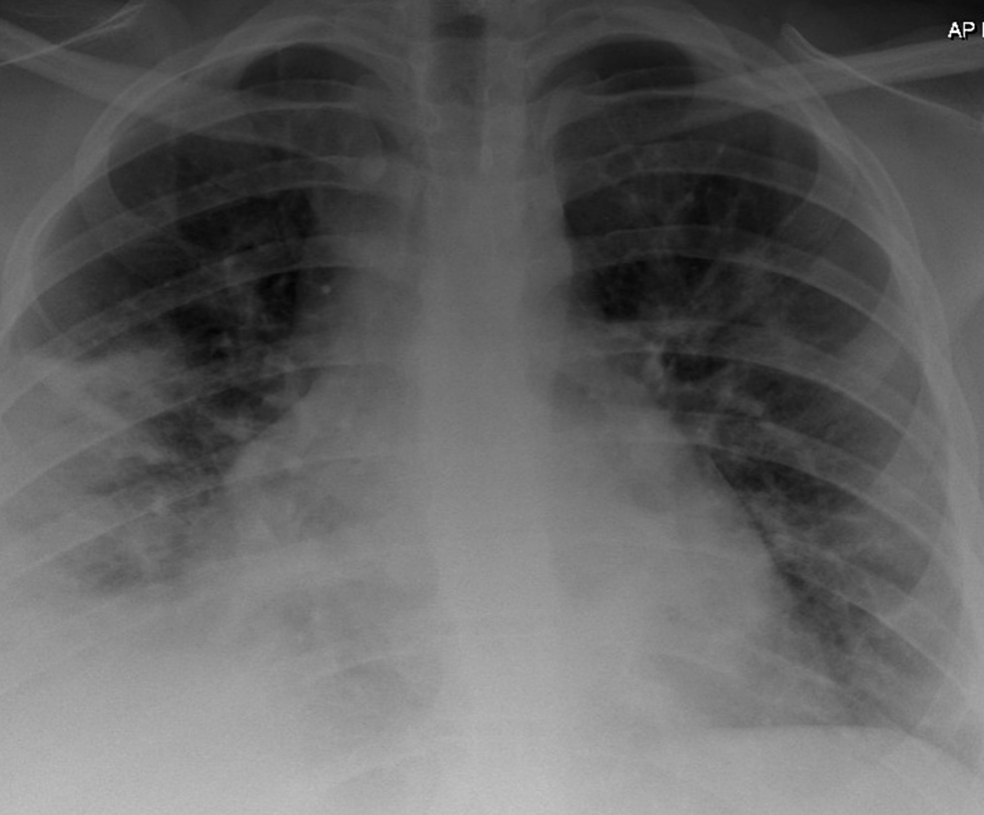
Chest X-ray showing COVID pneumonia

A 39-year-old female patient presented to the emergency department due to cough and fever with no significant history of comorbidities or risk factors. She had severe shortness of breath, requiring management in the high dependency unit (HDU) with high flow oxygen through a non-rebreather mask; she was prescribed antibiotics i.e. co-amoxiclav and clarithromycin. Her COVID-19 polymerase chain reaction (PCR) result came back positive. During admission, she received dexamethasone and tocilizumab. Chest X-ray showed features of COVID pneumonia (Figure 1). Her condition improved and she was discharged after six days.

She presented again on day 15, with left-sided facial droop, slurred speech, and left arm weakness. She also complained of severe headache with an intensity of 7 out of 10. Headache was sub-acute in onset; it was in the occipital region and bilateral, associated with dizziness. Clinical examination revealed left facial droop, nystagmus with fast component to the left, scanning speech, along with cerebellar signs on the left side including past pointing, dysdiadochokinesia, and abnormal heel shin toe test. Power was normal but Romberg's sign was positive and the patient failed to attempt tandem walk. She was admitted for further management. Her neurological examination was inconsistent but nevertheless showed cerebellar signs.

Her working diagnosis was CVT; initial blood investigations were normal except for D-dimer which was raised at 1875 ng/ml (Table 1).

**Table 1:** Blood tests on second admission with neurological symptoms WCC: white cell count; HB: hemoglobin; PLT: platelet count; INR: international normalized ratio; ALT: alanine aminotransferase; CRP: c-reactive protein; ALP: alkaline phosphatase.

WCC	6.2 x 10^9^/L
HB	14.3 g/dL
PLT	195 × 10^9^/L
Lymphocytes	2.71 × 10^9^/L
INR	1.0
D-DIMER	1875 ng/mL
ALT	80 IU/L
CRP	0.4 mg/dL
Sodium	140 mmol/L
Potassium	4.1 mmol/L
Urea	3.5 mmol/L
Creatinine	55 mmol/L
Adjusted Calcium	2.37 mmol/L
Albumin	38 g/L
Bilirubin	10 µmol/L
ALP	74 U/L

**Figure 2:**
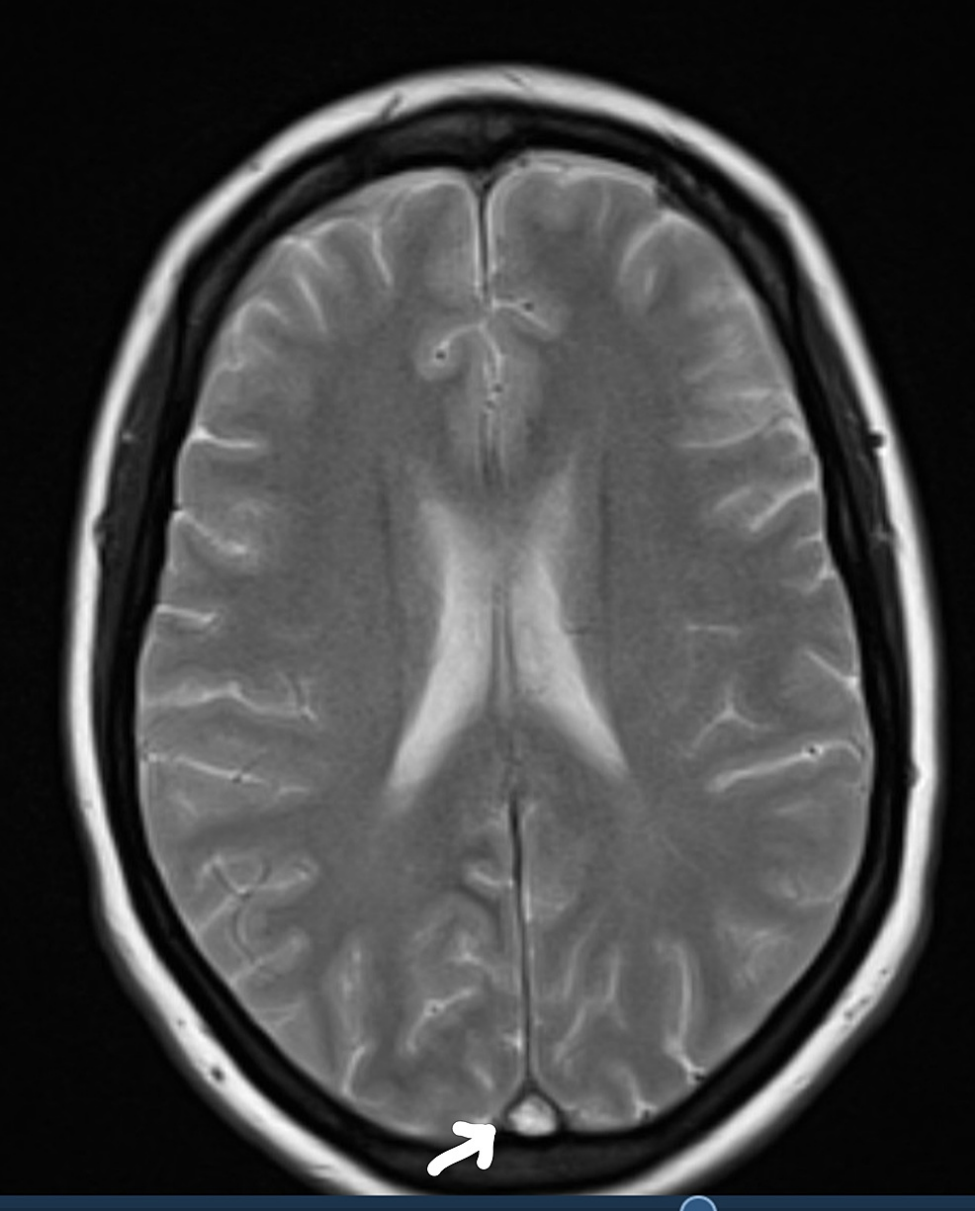
MRI head showing cerebral venous thrombosis

**Figure 3:**
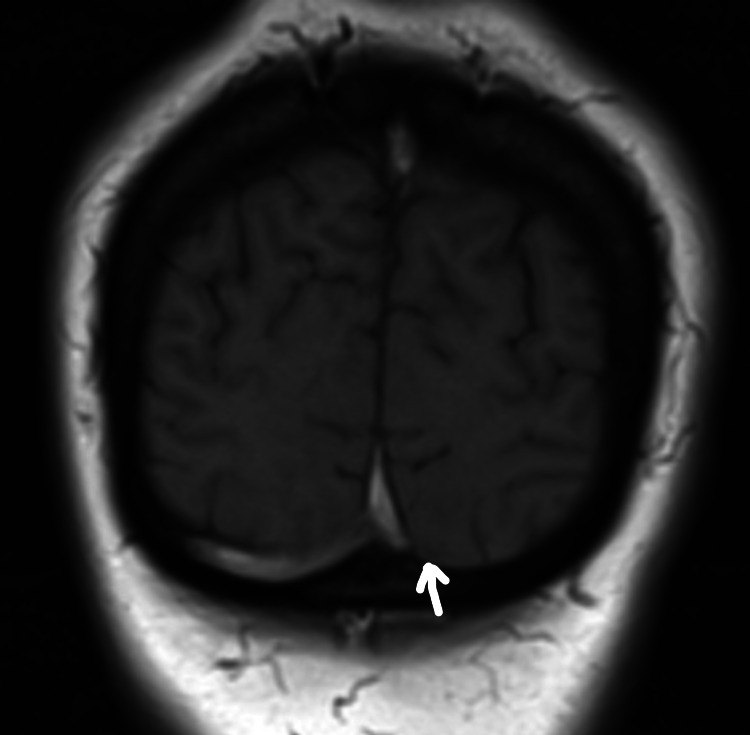
MRI head showing cerebral venous thrombosis

**Figure 4:**
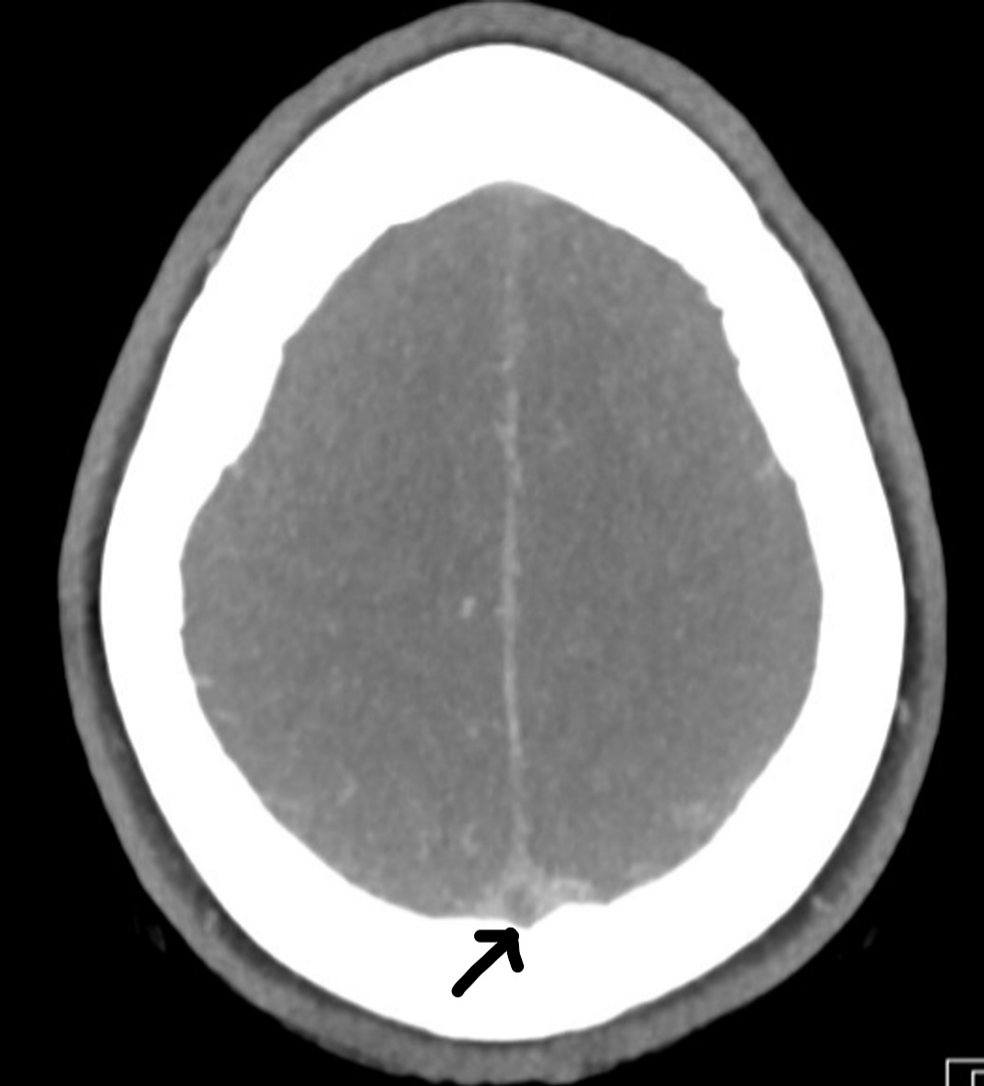
CT venogram showing reverse delta sign

**Figure 5:**
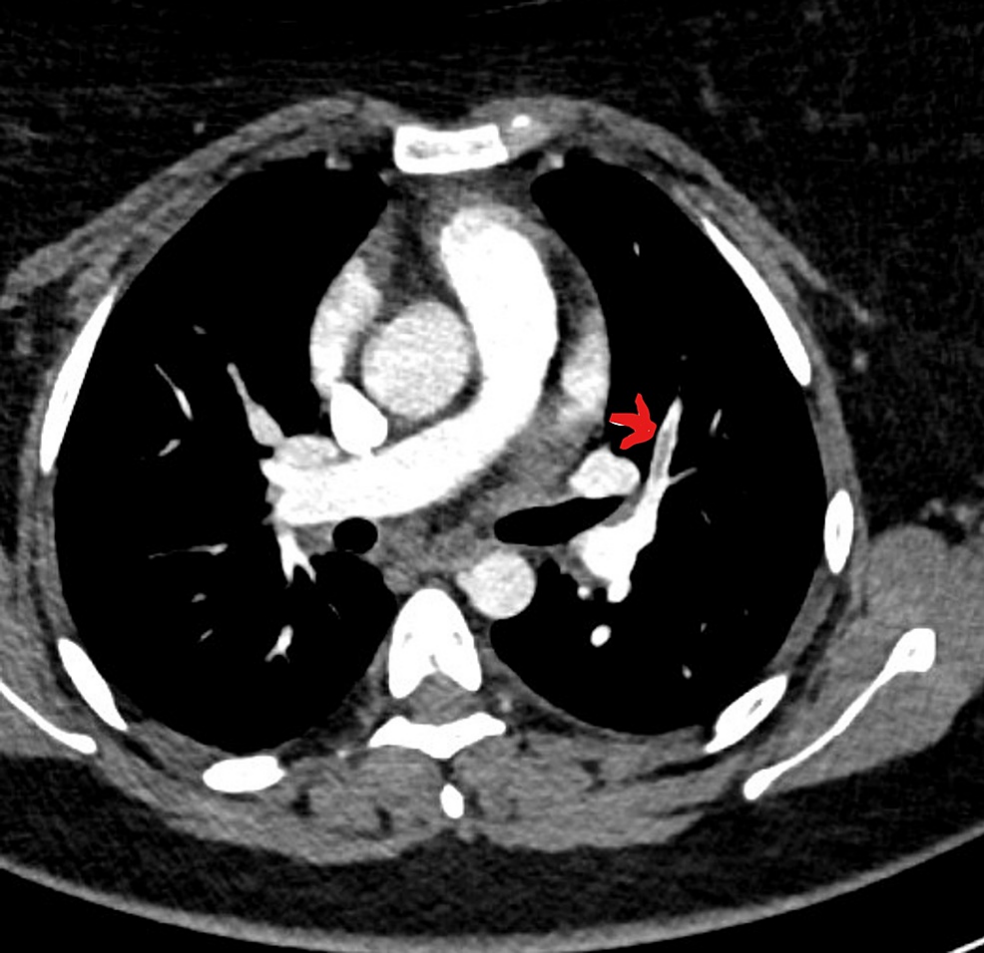
CT pulmonary angiogram (CTPA) showing segmental pulmonary embolism

She was investigated with head MRI and CT venogram showing evidence of thrombus in superior sagittal sinus with empty delta sign, small filling defects in the left transverse sinus, and inferior sagittal sinus not visualized properly (Figures 2-4). A filling defect was also seen in the anterior aspect of the straight sinus in the region of torcular Herophili, consistent with venous sinus thrombosis. She also underwent CTPA due to shortness of breath again, requiring supplemental oxygen (Figure 5). CT scan reported an isolated lingular acute segmental PE and patchy ground-glass shadowing throughout both lung fields confirming infective COVID-19 changes. She was started on a therapeutic dose of dalteparin (LMWH) and discharged on it. Since she made a good recovery, she was discharged eight days later with plans to follow up with the clinic after six months, along with a repeat MRI head and venogram. 

## Discussion

CVT is a relatively rare form of VTE, that accounts for approximately 1% of all forms of stroke. The common clinical presentation isolated intracranial hypertension syndrome, focal neurological deficits, and cavernous sinus syndrome. MRI with magnetic resonance venogram (MRV) is considered the gold standard for diagnosis. Anticoagulation with heparin or low-molecular-weight heparin is the mainstay of treatment. Endovascular management is indicated only when there are severe symptoms of CVT or worsening of symptoms despite anticoagulation therapy [10]. 

PE is the third most common cause of cardiovascular death worldwide after stroke and heart attack. Most PEs originate from DVTs of lower extremities, and approximately 50% of DVTs may lead to silent PE [11]. The truest explanation of the pathophysiology of VTE is Virchow’s triad i.e. stasis, endothelial disruption, and hypercoagulability [12]. The classic presenting symptoms are pleuritic chest pain (39%), dyspnea at rest (50%), and hemoptysis 20% [13]. CTPA has become the gold standard for diagnosis nowadays. Management of PE has evolved recently with the availability of local thrombolysis; mechanical extraction devices; hemodynamic support devices like extracorporeal membrane oxygenation; and surgical embolectomy.

Our case highlights the occurrence of CVT in a patient with a history of severe COVID-19 infection alongside PE and encourages keeping high suspicion of thrombo-embolism in such cases. We did a literature search to see the occurrence of CVT, PE, and COVID-19. We found substantial proof suggesting there is a predisposition for severe COVID-19 infection to trigger cytokine reaction which leads to a pro-inflammatory and pro-thrombotic state [14]. These patients may thus get afflicted from thromboembolic events, both venous and arterial [15]. A similar case like ours has been reported by Sugiyama et al. presenting with COVID-19 associated CVT and PE [16].

The presence of COVID-19 infection and consequent CVT seems to be a warning sign of a much more unfavorable prognosis than each condition separately. The known mortality rate of CVT is 15% and COVID-19 is 5.6%, but systematic reviews have found a much higher mortality rate in COVID-19 patients presenting with CVT, 40% by Baldini et al. and 45.5% by Tu et al. [9,17-19]. It is proposed that the possibility for developing this appalling outcome, in the reported COVID-19 associated CVT patients, may be due to the position of the obstructed sinuses, which indicates a greater predisposition for the deep venous sinuses to be occluded. Blockage in the internal cerebral veins or straight sinus was seen in 13% and 23%, respectively of the patients in the systematic review by Zicarelli et al. Interestingly, these deep sites of occlusion are generally infrequent and have a higher morbidity and mortality rate. Therefore, this can be a causative component of the increased mortality reported in these series, where almost 50% of the patients who died, had the inclusion of the deep cerebral veins [20,21].

This has steered to a number of recommendations being formulated for the use of anticoagulation in hospitalized COVID-19 patients, where those managed with anticoagulation were noticed to have better outcomes [14,22]. For the treatment of CVT, the first line of therapy remains the same, i.e. anticoagulation with low molecular weight or unfractionated heparin. There may be some benefit in advance commencement of anticoagulation in patients reckoned to have CVT or prone to its formation. Finally, thrombectomy should be considered as a viable therapeutic option in patients with CVT who have contraindications for anticoagulation [9].

## Conclusions

CVT is a serious complication of COVID 19. Clinicians should be aware of the clinical signs of CVT and make a prompt diagnosis. Treatment should be started early, seeing that the outcome of this predicament is fatal, if not treated adequately. Furthermore, in cases where high levels of D-dimer and fibrinogen are found, associated with neurological symptoms, imaging of the head should be performed to confirm the diagnosis. Anticoagulant therapy remains the mainstay of treatment. It is also noteworthy that data in the literature on this clinical scenario is not sufficient yet, requiring more robust and widely executed studies, evaluating the epidemiology, pathophysiology, diagnosis, management, and the outcome of the condition.
